# The Naturalist

**Published:** 1846-04

**Authors:** 


### THE NATURALIST.

This is the name of a periodical issued from Franklin College, near Nashville, Tennessee, by the Faculty of the College. The January number has appeared, and contains papers by Professors Loomis, Fanning, Fowler, and Eichbaum. It is to be published in monthly numbers of 48 pages, at $2 in advance, $2 50 if payment be delayed over three months, and $3 if delayed over six months. We tender to the editors our best wishes for the success of their enterprize, and run no risk in promising that its patrons will find it an instructive and valuable journal. We hope it will be sustained.

